# A high-throughput anaerobic method for viability assays

**DOI:** 10.1128/spectrum.02706-24

**Published:** 2025-03-05

**Authors:** Mohamed El-Fateh, Christian T. Meyer, Anushree Chatterjee, Xin Zhao

**Affiliations:** 1 Department of Animal Science, McGill University, Sainte-Anne-de-Bellevue, Quebec, Canada; 2 Department of Hygiene and Zoonoses, Faculty of Veterinary Medicine, Mansoura University, El-Dakhelia, Egypt; 3 Antimicrobial Regeneration Consortium (ARC) Labs, Louisville, Colorado, USA; 4 Chemical and Biological Engineering, University of Colorado Boulder, Boulder, Colorado, USA; 5 Molecular, Cellular, and Developmental Biology, University of Colorado Boulder, Boulder, Colorado, USA; 6 Sachi Bio, Colorado Technology Center, Louisville, Colorado, USA; Agriculture and Agri-Food Canada, Lacombe, Canada

**Keywords:** anaerobes, antibiotic resistance, susceptibility testing, bactericidal activity, bacterial viability, spores

## Abstract

Viability testing for anaerobes is a time-consuming and expensive process, posing challenges for research and public health settings. Here, we present a rapid, economical, and reliable method for testing anaerobe viability using the Geometric Viability Assay (GVA) with *Clostridium perfringens* as our model, a bacterium known for causing toxin-related systemic and enteric diseases. This method is efficient and cost-effective, requiring one pipette tip per sample, and is compatible with the economical anaerobic jar system. The results align with traditional plate-based assays in terms of colony-forming unit (CFU) measurements. Anaerobic GVA has low technical bias and a dynamic range extending over 5 orders of magnitude. In addition, our method determined the bactericidal activity of antibiotics in a dose-dependent manner, when an antibiotic sensitivity testing (AST) was performed with a panel of four antibiotics (ampicillin, gentamicin, meropenem, and tetracycline). Furthermore, the minimum concentrations for complete bactericidal activity (MBC) of four clinical isolates were determined and the MBC concentration for tetracycline was up to 8× higher than the concentration for complete growth inhibition (MIC). Additional tests involving *Clostridium bifermentans* and *Clostridium sporogenes* demonstrated the generality of our method for other anaerobic species. Beyond viability testing, the GVA measured spore concentrations of various *Clostridium perfringens* isolates, showing consistency with classical plating methods. Our study confirms that the anaerobic GVA is a valuable tool for rapid, accurate viability screening in anaerobic settings and is compatible with routine assays, such as AST and spore screening. This method enhances the scalability and utility of anaerobic viability-based assays.

**IMPORTANCE:**

The routine assessment for the viability of anaerobes is based on bacterial plating, but so far, it has been limited in throughput by the long preparation steps and the tedious anaerobic culturing. Thus, comparatively little is known about the susceptibility pattern, and the sporulation of anaerobes because of the absence of the proper method. Here, we show GVA can quantify the anaerobic *Clostridiums* colonies accurately by utilizing an anaerobic jar to measure viable cells and spores in high throughput with minimal volumes of reagents and at a comparable time to the traditional viability testing practice. Furthermore, this method enabled high-throughput detection of the bactericidal activity of the antibiotics against anaerobes and allowed for the quantification of hetero-tolerant/resistant subpopulation, which was previously unattainable. Our approach is rapid and easy to use, making it ideal for various applications where high-throughput capabilities can drive innovation, including drug-microbe interactions, host-microbe interactions, and microbe-microbe interactions.

## INTRODUCTION

Anaerobic bacteria represent a significant constituent of the human and animal microbiota that inhabit mucosal membranes. Infections caused by a subset of these opportunistic pathogens can be serious, leading to high mortality rates (1). For example, *Clostridium perfringens* is a gram-positive, anaerobic, spore-forming bacillus commonly found in soil, food, dust, and the intestinal tracts of humans and animals (2). Toxins of *C. perfringens* cause different diseases in both humans and animals, ranging from subclinical manifestations to serious diseases (3). According to the CDC, *C. perfringens* ranks second among the identified etiological agents of food-borne illness in the US with an estimated 1 million cases annually (4), and the results in approximately $384 million of economic loss (5). *C. perfringens* is found in high-protein foods, such as raw meat and poultry (6). *C. perfringens* can proliferate rapidly in food, doubling in as little as 10 minutes (7) and create durable spores that can endure high temperatures, intense pressure, harmful chemicals, food preservatives, and even radiation (8, 9). Furthermore, *C. perfringens* enterotoxin and treatment with broad-spectrum antibiotics causes non-food associated diarrhea (10) but death due to diarrhea caused by *C. perfringens* is uncommon. Other conditions, such as necrotic enteritis in animals, enterocolitis, myonecrosis (gas gangrene), and septicaemia with intravascular haemolysis can occur due to *C. perfringens* infection (11). Thus, the significance of accurately and rapidly detecting *C. perfringens* cells and spores in food and clinical samples cannot be overstated to ensure food safety for consumers and proper infection diagnosis.

Culture-based approaches to measuring bacterial viability play an integral role in profiling and characterizing different anaerobes ranging from food safety (12, 13), drug discovery (14–17), environmental public health (18), and applied microbiology (19–21). The standard method for anaerobic viability testing is the agar-plate CFU assay. However, this approach is labor-intensive and time-consuming and requires a considerable amount of consumables such as agar plates, solid media, broth media, dilution reservoirs, and plastic spreaders (22). Alternative approaches should be developed.

Antibiotic sensitivity testing (AST) is a crucial routine in diagnostic and research laboratories to determine the effective antibiotic concentration and profile the resistance pattern of bacterial isolates (23). Inadequate AST impacts the infection prognosis as selecting improper antibiotics increases mortality (24, 25). AST generates the minimal inhibitory concentration (MIC) value by assessing broth turbidity optically or/and the minimum bactericidal concentration (MBC) by plating the treated culture to test for the viability of treated bacteria following drug exposure (26). For anaerobes, due to the complexity of anaerobic culturing and the lack of standard methods, screening for MBC is limited. However, such information is necessary when testing new antibacterials, and when the bacteriostatic concentration is not enough to eliminate the infection (27). Assays relying solely on optical density (OD) such as broth microdilution are not clinically standardized for all anaerobes (including *Clostridia*) due to the inconsistent results compared with the referenced plate-based agar dilution method (28). Moreover, OD value does not necessarily correlate with the absolute bacterial count (29). Besides, the intrinsic color of the testing drugs may affect the optical interpretation (30). For spore-forming, as well as toxin-producing anaerobes, it is important to check the bactericidal efficacy of an antibiotic, as the spore-forming bacteria with no visible growth can be falsely reported as susceptible (31–33). Thus, a high throughput assay to quantify the activity of an antibiotic would substantially improve the resolution of identifying the hetero-tolerant/resistant bacterial subpopulations in AST testing (34, 35).

The most common systems to cultivate anaerobes are the anaerobic jars or anaerobic chambers (36). The anaerobic chambers, also known as glove boxes, provide room for large-scale culturing; however, this comes at the expense of high maintenance costs, advanced training, large space requirements, and limited mobility. The jar system offers a more convenient culturing apparatus in terms of portability, size, and simplicity in CO_2_ generation, but its size limits the number of anaerobic samples that can be examined (37). Therefore, there is an outstanding need for high-throughput, low-cost measurement of viability for anaerobes.

Counting bacterial spores is a critical component in microbiological research and industrial applications, as it provides essential insights into bacterial viability, sporulation efficiency, and the potential for pathogen transmission (38). Current methodologies for spore counting, such as traditional plate counts and microscopic examination, are often labor-intensive, time-consuming, and subject to significant variability and human error (39, 40). These methods typically fail to accommodate the high-throughput demands of contemporary microbiological studies, especially when assessing anaerobic bacteria (41, 42). Therefore, there is a pressing need for a new, robust, and high-throughput technique that can accurately quantify spore viability under anaerobic conditions. Such advancements would not only enhance the precision and efficiency of microbiological assays but also facilitate the development of novel antimicrobial strategies and improve our understanding of microbial ecology in diverse environments.

Recently, a viability assay, called the Geometric Viability Assay (GVA) has been developed (43). GVA utilizes the axial position of colonies grown in a pipette tip to calculate the concentration of viable cells in a sample. The probability of a colony forming is lower at the apex of the cone as opposed to its base, due to differences in the cross-sectional area. By measuring the position of several colonies within a subvolume of the cone and employing the corresponding probability of colonies’ location, one can achieve a reliable estimation of the total colony count throughout the entire cone. The assay was previously developed for aerobic bacteria, and it has not been deployed to study anaerobes. Herein, we extended the protocol for the GVA to develop a high-throughput method to measure the viability of anerobic bacteria, using *Clostridium* as a model. Our method matches the high-throughput capacity of 96-well plates. By reducing the footprint of a sample to a single pipette tip, the viability of 96 samples could be tested in a single anaerobic jar, saving time, space, and reagents.

## RESULTS

### Establishment of anaerobic GVA protocol to measure *Clostridium* viability

**Fig 1:**
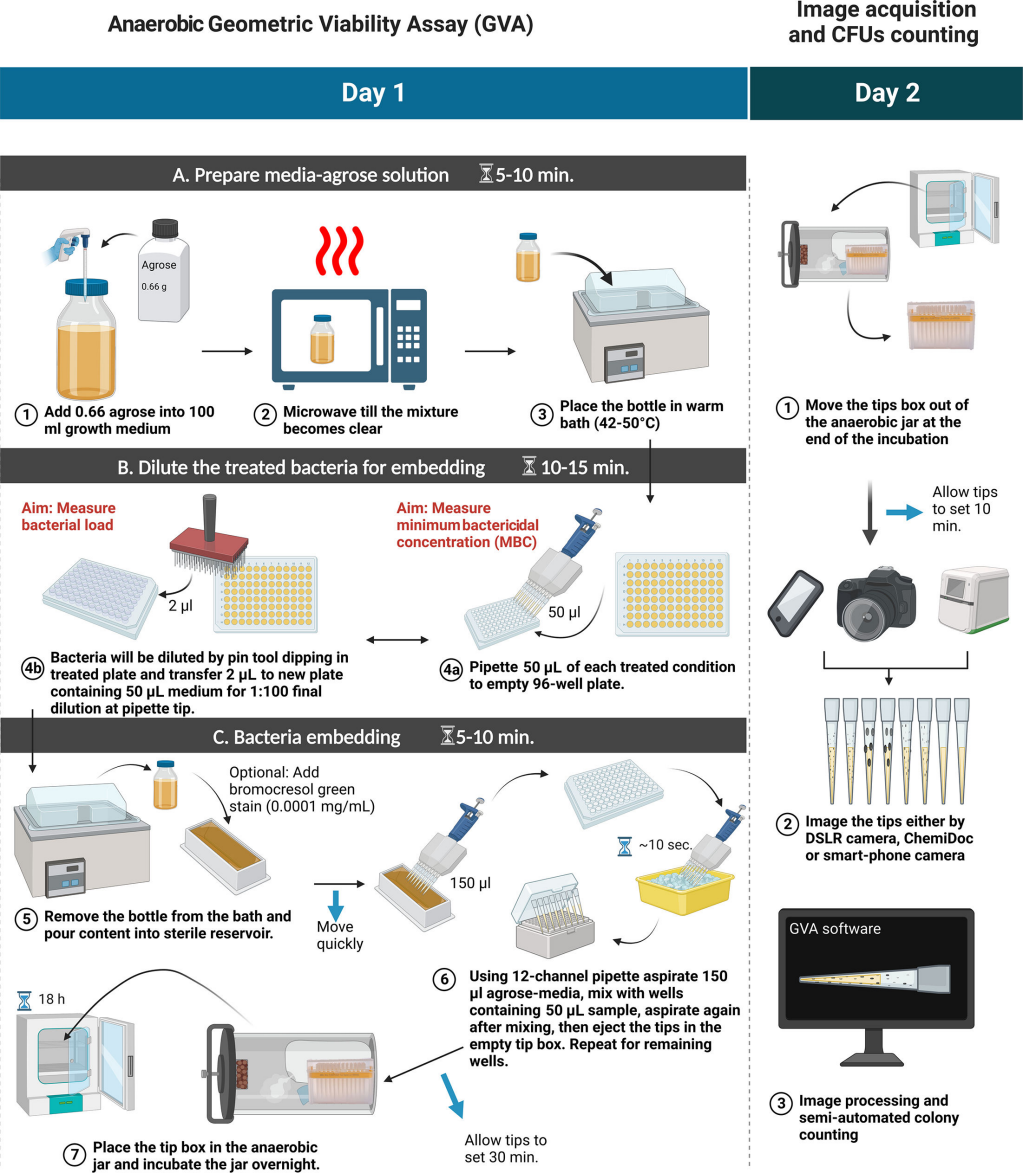
Illustration of the GVA for measuring the viability of *Clostridium* using the anaerobic jar system. On day 1, GVA is conducted to check the viability of anaerobe following the treatment. GVA comprises three steps: the first step is to prepare the media-agarose mixture; the second step is to prepare the treated bacteria for embedding; and the third step involves mixing the diluted samples with the embedding solution and allowing the agarose to solidify inside the pipette tip. Then, the tip box containing 96 samples is placed into the anaerobic jar and incubated overnight. On the second day, the tips are retrieved from the jar for imaging and counting the colonies.

**Fig 2:**
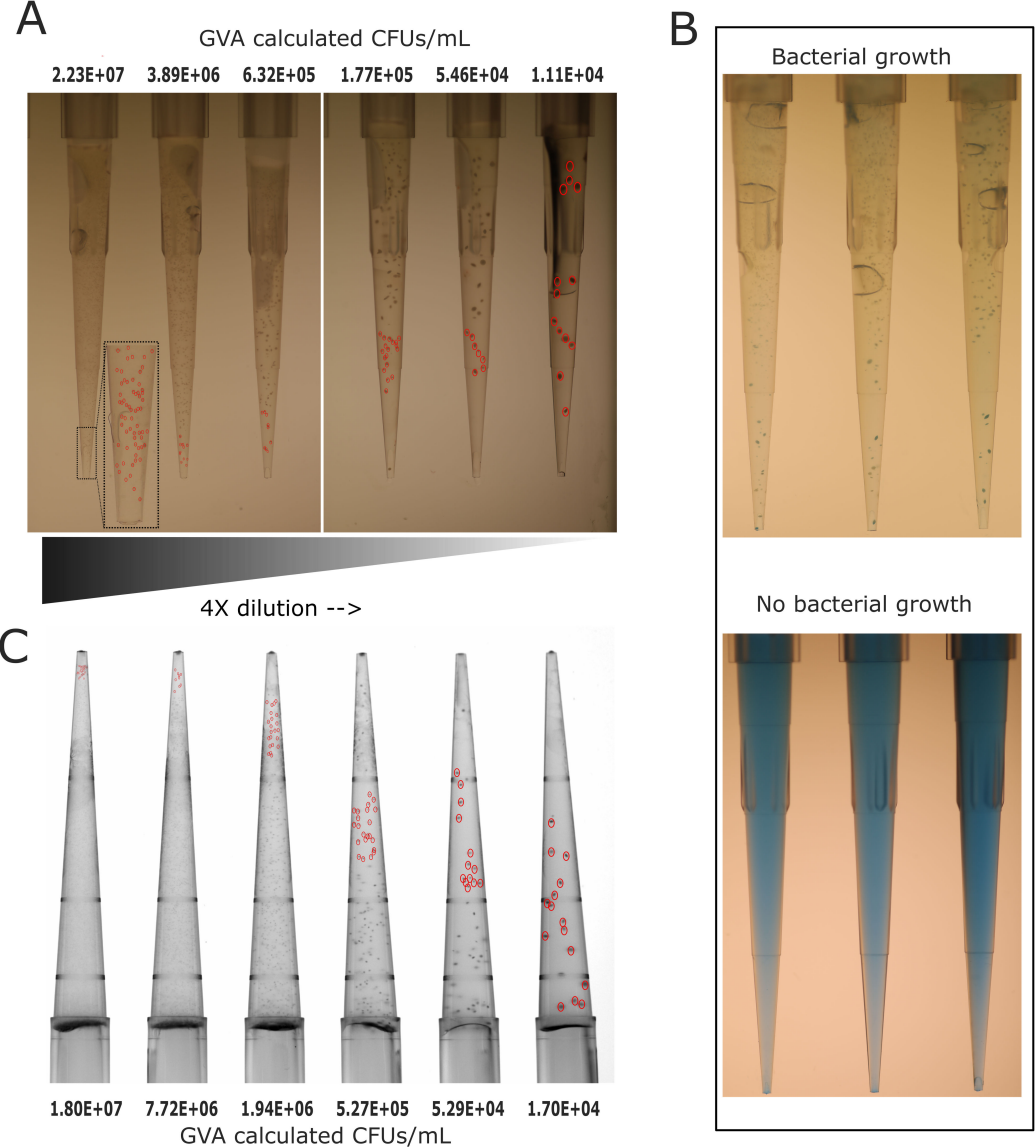
GVA quantification of *C. perfringens* colonies embedded within the pipette tips. (**A**) Dilution series of *C. perfringens* embedded in 150 µL TGB with 0.5% agarose in P200 pipette tips. GVA calculation (top) based on the position of colonies circled in red. Images acquired with the mirrorless camera system. (**B**) The visual difference between tips with and without bacterial growth when 0.0001 mg/mL BG was added to the TGB-agrose mixture. (Top) The bacterial colonies are stained blue with a clear background. (Bottom) The image showed no bacterial growth resulting in tips with homogeneous blue embedding. (**C**) GVA pipette tips containing *C. perfringens* colonies imaged using White *Trans* illumination mode of ChemiDoc with 590/110 filter. GVA calculation (bottom) based on the position of colonies circled in red.

We adapted the GVA method to measure *Clostridiums’* viability by fitting the sample-containing pipette tips into an anaerobic culturing jar system (Fig. 1). The GVA itself involves three steps: (i) preparing the embedding solution by melting the agarose at 0.66% (wt/vol) in thioglycollate broth (TGB) media and cooling to 50°C; (ii) diluting the bacterial samples into a 96-well plate such that the maximum CFUs/mL expected is <10^7^; and (iii) mixing the diluted samples with the embedding solution and allowing the agarose to solidify inside the pipette tip. The overall process takes approximately 30 min for one 96-well plate, and an additional 5 min for subsequent plates. The whole box of GVA tips was then placed in an anaerobic jar and incubated overnight. After incubation, the colonies were clearly visible inside the pipette tip (Fig. 2A). For 96 viability measurements, GVA requires one P200 pipette tip box, 20 mL growth media (plus 25 mL if an initial dilution is required), one reservoir, and one 96-well plate. Thus, our approach requires fewer consumables than the traditional agar counting method.

The original GVA protocol recommends adding Triphenyltetrazolium chloride (TTC) to increase colony contrast for facultative aerobes; however, TTC did not stain *C. perfringens* colonies. In trying other stains, we found the pH-sensitive dye Bromocresol Green (BG) to stain the bacterial colonies blue against a slightly colorless background (Fig. 2B). In contrast, tips containing no bacterial colonies are homogeneously blue (Fig. 2B). Adding BG is optional, but using it, offers enhanced visibility. Additionally, BG allows a qualitative readout of the drug MBC with the naked eye. MBC determination does not require the bacterial dilution step, further shortening the GVA by directly embedding the sample into the tips.

To image the pipette tips, we initially utilized the previously described mirrorless camera system (43). In this study, we also explored the utility of a GelDoc/ChemiDoc imaging system for detecting colonies (Fig. 2C). Optimal visualization was achieved by positioning a set of 12-tip (held by 12-channel pipette) on the white tray and capturing the tips using White *Trans* Illumination mode with 590/110 filter (Fig. 2C). To make the GelDoc images compatible with the current GVA software, a custom MATLAB script was developed (see materials and methods section).

### GVA viability measurements are correlated with spot plate-based estimates for *Clostridium* species

**Fig 3:**
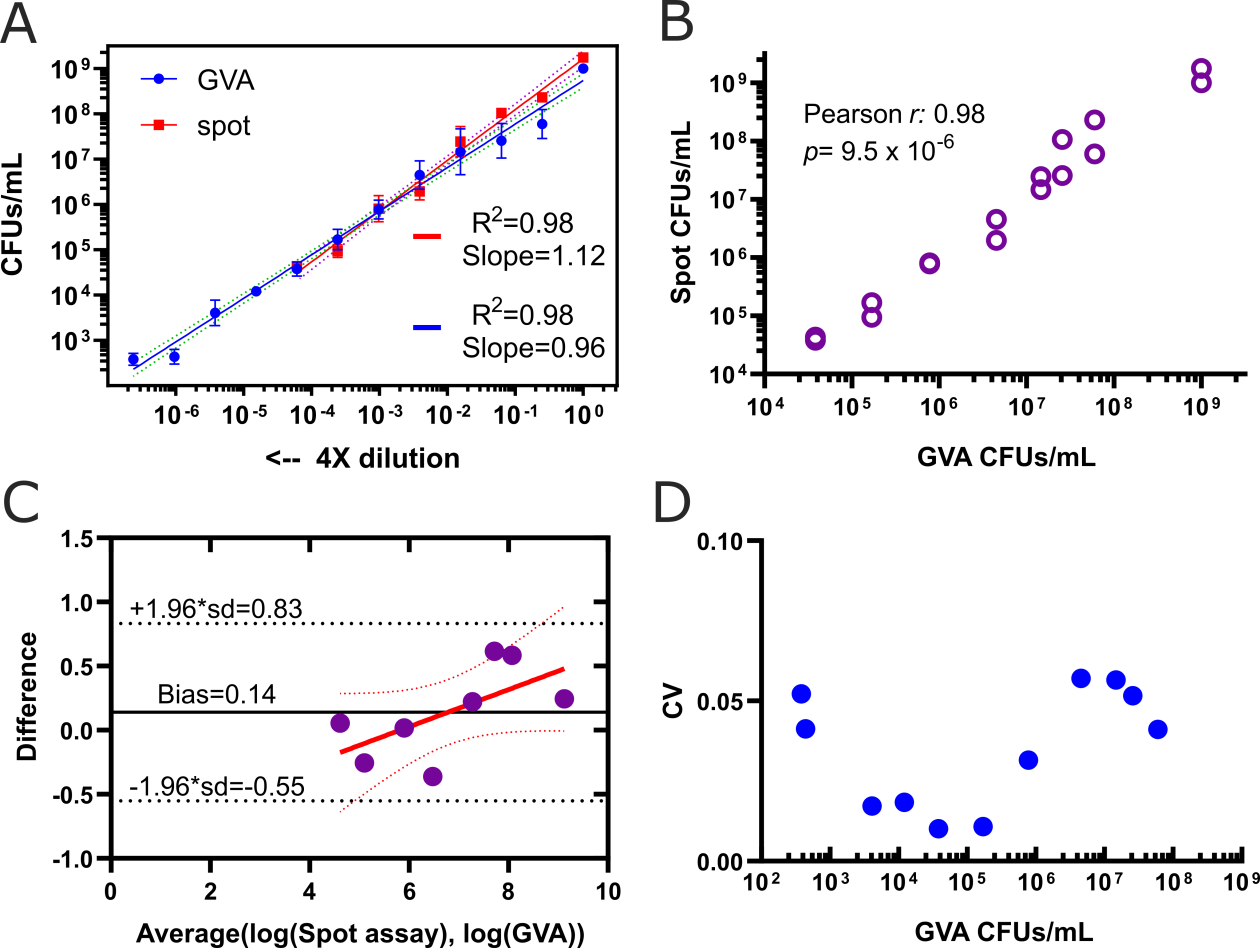
GVA quantified Clostridium CFUs with an extended detection range. (**A**) *C. perfringens* CFUs/mL calculated using spot assay (red squares) GVA (blue dots) for a 4× dilution series from an overnight culture. Data points are the mean of 5 replicates for GVA and three replicates for spot assay. Mean calculated after taking the log10 of CFUs/mL. Blue and red lines are the linear regression fit to the dilution series for GVA and spot CFU assay, respectively. It is expected that a slope of 1 will be observed on a log-log plot if the GVA estimate scales linearly with the dilutions. (**B**) Pearson correlation between spot CFU and GVA for all dilutions where colonies could be counted using both. The calculation of the correlation coefficient was performed using the logarithmic values. (**C**) Bland-Altman plot comparing GVA and spot assay CFU measurements. CFU values were log-transformed for the comparison. The method difference (GVA-Spot assay, y-axis) as a function of the average of the methods (x-axis) was fitted using a linear regression model (red line) with confidence intervals depicted (dotted red lines). The limits of agreement, equal to 1.96× the standard deviation in difference between the methods is depicted in the black dotted lines. The bias, equal to the mean difference across the data, is annotated in the solid black line. (**D**) Coefficient of variation (CV) among five technical replicates for various *C. perfringens* concentrations was calculated using GVA. Technical replicates were used to determine the noise in GVA quantifications.

We tested the ability of GVA to reliably quantify the CFUs/mL of a serial dilution of the overnight *C. perfringens* CP1802 culture. The CFUs/mL of the same samples were also determined using the traditional spot assay method. GVA detected the viability of a 4× dilution series of *C. perfringens* linearly across 5 orders of magnitude (Fig. 3A, blue line, slope = 0.96). GVA’s dynamic range was 2 orders of magnitude greater compared with the spot assay (Fig. 3A, red line). The viability measurements between the two methods were significantly correlated (Pearson *r* = 0.98, *P* = 9.5 × 10^−6^) (Fig. 3B). The Bland-Altman test indicated that the bias between spot assay and GVA viability measurements was 1.38 (10^0.14^) across 4 orders of magnitude (Fig. 3C), and the trend-line slope of the method’s difference as a function of the CFUs/mL was not significantly different from zero (*P*-value = 0.092). Therefore, GVA and spot assay can be used interchangeably.

The technical noise of GVA viability measurements was computed among four technical replicates. The coefficient of variation (CV) among replicates was used to calculate noise. The GVA noise was found to be ≤5% across all measured CFU concentrations (Fig. 3D) and was heteroskedastic, as previously reported (43). In addition, the TGB media outperformed TSC media for *Clostridium* when imaging with GelDoc due to the diffusion of the reduced iron sulfide in the pipette tip obscuring other colonies (Fig. S1A and B). The colonies could be identified when imaging with the previously described imaging configuration (43) (Fig. S1C). Additional testing with *Clostridium bifermentans* and *Clostridium sporogenes* by GVA was also successful in reliably quantifying their viability across the bacterial serial dilutions (Fig. S2).

### GVA screened the spore-formation of *C. perfringens*

**Fig 4:**
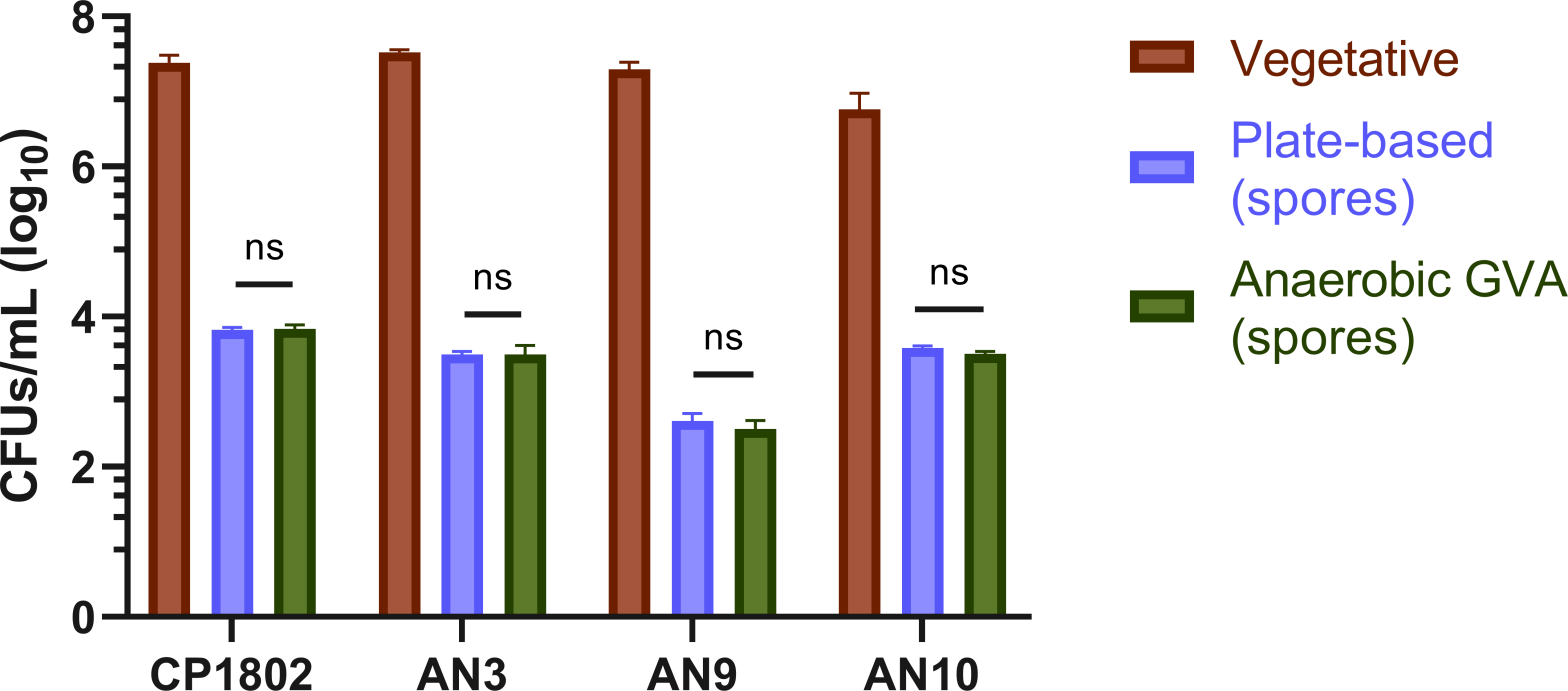
Vegetative cell count and comparison of spores count between plating on BHI (standard method) and anaerobic GVA. The *C. perfringens* strains were grown in TGB medium supplemented with 0.01% theobromine overnight at 37°C. A sample from the culture was taken to count the vegetative cells. The overnight cultures were then heat shocked for 10 min at 75–80°C. Aliquot from heat-shocked cultures were then plated onto BHI agar plates and grown overnight at 37°C for colony counting. Another aliquot was quantified using GVA anaerobic protocol. ns, *P* > 0.05 (Student’s t test, compared with plate-based counting). *P* value adjusted for multiple comparisons using Bonferroni method. All experiments were repeated three times, and mean values (log10 scale) are shown. The error bars indicate standard deviations.

Using GVA, we investigated the spore-formation ability of four *C. perfringens* clinical isolates (CP1802, AN3, AN9, and AN10). The vegetative cells were quantified, prior to the heat shock, using the GVA method. Then, after the heat shock, the cultures for the spore formation were screened, using both the plating method and the GVA for the same sample (Fig. 4). No significant differences were found between plate-based and GVA CFUs quantifications for the four tested isolates.

### GVA quantified the dose-dependent bactericidal effect of four antibiotics against a *C. perfringens* clinical isolate

**Fig 5:**
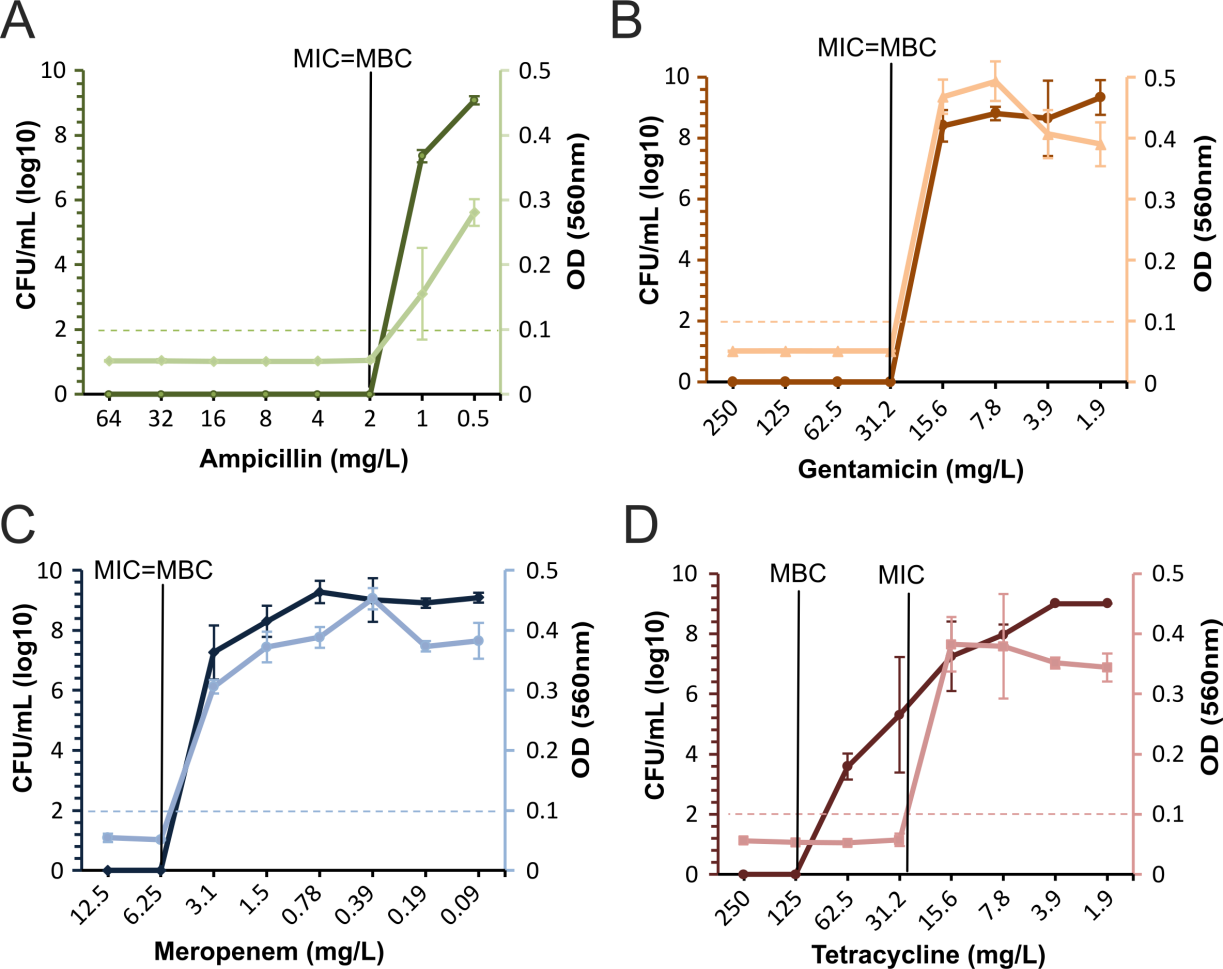
*C. perfringens* viability in response to the antibiotic treatment. The efficacy of (**A**) ampicillin, (**B**) gentamicin, (**C**) meropenem, or (**D**) tetracycline in reducing survival or growth of CP1802 after 20 h at 37°C anaerobic condition. Darker lines show the dose-dependant viability of *C. perfringens* at each antibiotic concentration measured by GVA as a log_10_(CFUs/mL). The lighter lines depict the OD of treated wells. The dotted horizontal lines represent the threshold OD values for determining the MIC. The black vertical lines show the MIC, MBC, or both values. The points represent the mean from three biological replicates, and the error bars indicate the standard deviation between replicates.

We further investigated the GVA’s utility in measuring *C. perfringens* viability under various antibiotics. We quantified the *C. perfringens* clinical isolate (CP1802) viability following 20 h of treatment with a panel of four antibiotics: ampicillin, gentamicin, meropenem, and tetracycline (Fig. 5). The MBC values were compared with the MIC values determined from optical density (OD) measurements after 20 h treatment. Although MIC and MBC were equal for ampicillin, gentamicin, and meropenem (Fig. 5A through C), the MBC and MIC were different for tetracycline by a factor of four, at 125 and 31.2 mg/L, respectively (Fig. 5D). GVA reveals that *C. perfringens* cells capable of surviving 2× the MIC of tetracycline for 20 h occurs at a rate of 1:100,000. These rare cells can only be observed using a viability assay with a large dynamic range, such as GVA. Although the correlation coefficient between the OD and viability measurements was high (*r* = 0.847), the absolute correlation was low due to the smaller dynamic range of the optical density.

### GVA screened the minimum bactericidal concentration (MBC) of antibiotics against four clinical *C. perfringens* isolates

**Fig 6:**
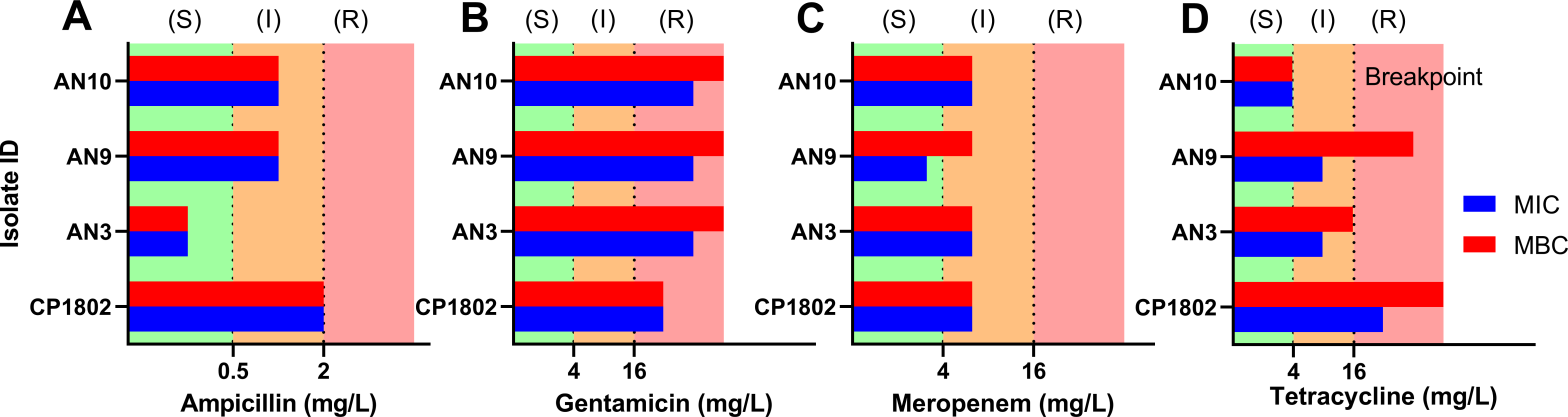
MICs and MBCs of panel of four antibiotics (**A**) ampicillin, (**B**) gentamicin, (**C**) meropenem, and (**D**) tetracycline against four clinical isolates of C. perfringens. MIC: The minimum inhibitory concentration of antibiotic where no bacterial growth was observed after 20 h. MBC: The lowest concentration of antibiotic that is bactericidal after 20 h treatment. Resistance classification is based on (44). These data represent the results from three biological replicates. R, resistant; I, intermediate; S, susceptible.

Following the successful application of GVA in MBC determination in CP1802, we expanded to profile three additional *C. perfringens* clinical isolates (AN3, AN9, and AN10) with our panel of four antibiotics. In total, we measured 384 viability conditions, utilizing only 77 mL liquid media and four tip boxes. Although each strain showed different MBC values to each antibiotic, the resistance interpretation differs across the panel (Fig. 6). All strains were resistant to gentamicin. With the exception of CP1802, all tested strains displayed sensitivity to ampicillin. All strains showed intermediate susceptibility to meropenem, except AN9. For tetracycline, AN3 and AN9 isolates showed intermediate susceptibility, whereas CP1802 was resistant and AN10 was sensitive. The AN3 strain was the most susceptible to ampicillin, with MBC and MIC values of <0.5 mg/L. In the three added clinical isolates, the MBC values were twice the MIC values for gentamicin. The meropenem MIC and MBC values were the same in all tested strains except AN9, where the MBC was higher by 2-fold. As with CP1802, the MIC and MBC values for AN3 and AN9 were not equivalent for tetracycline. In particular, the MBC was 8-fold higher than the MIC for the AN9 strain.

## DISCUSSION

The significance of accurately detecting and quantifying *C. perfringens* in food, environmental, human, and animal samples cannot be overstated, considering its status as a prominent cause of different diseases in both humans and animals. *C. perfringens* viability is conventionally assessed using the plate spread assay. Although widely used, the plate spread assay is plagued by labor-intensive procedures, prolonged timelines, and substantial resource requirements, including agar plates, solid and broth media, dilution reservoirs, and plastic spreaders. Other approaches to check for anaerobes viability have pros and cons (Table S1). Nevertheless, none of the mentioned methodologies combines the simplicity, affordability, dynamic range, and versatility in anaerobic viability assays. The complexity of anaerobic culturing adds additional challenges in performing high-throughput viability studies. Currently, anaerobe AST measurements are infrequent in the clinic. The ability to screen for novel compounds in research settings by high-throughput methods could accelerate the development of novel therapeutics.

Antibiotic susceptibility testing (AST) for anaerobic bacteria is necessary to predict the response of clinical infections to antibiotics (45–47). Numerous limitations inherent in currently available anaerobic AST assays compromise their practicality. There is a pressing need for a readily available methodology that offers ease, affordability, and flexibility in routine AST of anaerobic bacteria. Determination of the MBC through viability assays has microbiological importance in detecting hetero-resistance, testing drug potency, and assessing whether the drug is bacteriostatic or bactericidal (48). Typically, if the MBC is significantly higher than the MIC (usually MBC/MIC ratio >4), the drug is considered bacteriostatic. This indicates that the drug inhibits bacterial growth at low concentrations but requires much higher concentrations to kill the bacteria. However, if the MBC is close to the MIC (usually MBC/MIC ratio ≤4), the drug is considered bactericidal. This indicates that the drug can kill bacteria at concentrations close to those required to inhibit their growth.

We present a high-throughput, economical protocol to check the viability of *Clostridium* anaerobic bacterium in this study. Our approach harnesses the advantage of the anaerobic jar system in conjunction with the Geometric Viability Assay (GVA). Our method showed an accurate and robust quantification of 96 samples in one anaerobic jar. The GVA relies on the shape of the cone to run the dilution *in situ*, thus avoiding the dilution step, which is the most laborious step in the plate spread assay. Explicitly, the volume at the tip of the cone is much less than at the base, which results in colonies being more likely to form at the base than at the tip. Thus, by utilizing the intrinsic information in the geometry of the cone, the CFUs could be enumerated by calculating the distance between the colonies and the tip of the cone.

We chose *C. perfringens* as an anaerobic model for anaerobes viability screenings. Using our approach, *Clostridium* CFUs were quantified across a large dynamic range with low errors and the colonies were clearly distinguishable (Fig. 2A). BG was adopted in this study, which stained *C. perfringens* colonies blue. Furthermore, BG staining helped to differentiate the densely contained pipette tips from the tips with no colonies (Fig. 2C). By using the perfringens selective agar as an embedding media, it is possible for direct quantification and identification of *C. perfringens* in a single assay. Our method had a larger dynamic range compared with the plate spread assay (Fig. 3C). This ability is attributed to the large culture volume (50 µL) used in GVA compared with only 5–10 μL (droplet) in the case of plate spread assay, this corresponds to lowering the limit of detection by 10-fold. Our approach requires fewer consumables compared with traditional viability assays, which rely on a dilution series. For 96 viability measurements, GVA required one P200 pipette tip box, 20 mL growth media (plus 25 mL if requiring an initial dilution), one reservoir, and one 96-well plate. In contrast, 96 measurements with the routine dilution approach require 2,400 mL growth media and cost around 4–6 h (26). Using our approach, we quantified the dose-response of the *C. perfringens* and characterized various MIC/MBC patterns, which varied among the tested antibiotics (Fig. 5), and the different strains tested in this study (Fig. 6). This highlights the importance of performing viability assays in the context of increasing the bacterial resistance where the isolates susceptibility pattern is unpredictable (49). Moreover, bacterial response to an antibiotic has different forms, such as hetero-tolerant/resistant, where subpopulations of bacterial cells have the ability to survive the inhibitory effect of the antibiotic (34, 50). The gold standard to measure these phenomena is population analysis profiling (PAP). This method entails performing the standard MIC determination with 2-fold antibiotic increments and by use of spread plate techniques for CFU counting at each concentration (51). However, there is no standard to measure the hetero-resistance/tolerance in anaerobes, due to the lack of proper methodology (48). Employing anaerobic GVA holds significant potential to detect the heterogeneity subpopulation in response to the added antibiotic. The disparity between the MIC and MBC of tetracycline may be attributed to hetero-resistance within the sample, resulting in rare cells surviving treatment at concentrations higher than the MIC (Fig. 5). Recently, P. Müller et al. (30) developed a high-throughput screening for anaerobes based on micro-dilution AST in 96-well plates. However, the readout was limited to OD values, without differentiating the MBC from MIC. Our protocol enables the detection of the viable anaerobe in high throughput to screen for the bactericidal concentration, expanding it to quantify the number of viable cells post-treatment and capturing a higher resolution AST measurement.

We report a new imaging approach employing GelDoc/ChemiDoc instruments. Previously, Meyer et al. (43) reported three imaging options, including a DSLR camera, iPhone 12 (with macro lens), and paper-based GVA. These options offer varying degrees of resolution, simplicity, and cost. Herein, we tested the utility of the GelDoc/ChemiDoc for imaging the tips. The GelDoc/ChemiDoc imaged the tips at high resolution where the colonies were distinguishable, enabling 5 orders of dynamic range in a single pipette tip. These instruments are widely available in laboratories, working on microbiology and molecular biology. Hence, integrating the GelDoc/ChemiDoc imaging power in GVA will increase GVA technology accessibility, increasing its adoption in various laboratories.

There are some limitations to this method, particularly in the step of imaging and colony counting. Although there is an established pipeline for image acquisition and colony counting, including semi-automated colony segmentation, it takes ~30 min for 96 samples, representing the most time-consuming step compared with the sample preparation and embedding steps. Moreover, the three-dimensional nature of the colonies makes it challenging to assess their morphology, particularly at high densities, potentially limiting the ability to use morphology to identify bacterial strains in elective media. We performed our method in the common 2.5 L anaerobic jar; however, there are larger jars such as AnaeroPack rectangular Jar with 7 L volume. This would increase the capacity of the number of tips that could be incubated in the jar. The performance of GVA in AST assays, and its concordance with other anaerobic AST methodologies, warrants further investigation using a broader range of clinical isolates. Overall, our protocol substantially reduces the time, materials, and space requisite for anaerobes viability assays, compared with the routinely used plate-based assay. Furthermore, its compatibility with the universal anaerobes culturing jars allows for high-throughput viability screening of anaerobes. This method has the potential to be applied in the human, animal, environmental, and food microbiology sectors.

## MATERIALS AND METHODS

### Bacterial isolates and culture condition

Four clinical *C. perfringens* isolates were used. The CP1082 strain was obtained from Prof. Martine Boulianne’s laboratory at Université de Montréal. It was recovered from chickens with Necrotic Enteritis disease. The remaining three *C. perfringens* isolates (AN3, AN9, and AN10) and other clostridium species (*Clostridium bifermentans* and *Clostridium sporogenes*) of poultry origins were obtained from Prof. Jennifer Ronholm’s laboratory at McGill University. From frozen stock, each isolate was grown in Thioglycolate broth (TGB) (Thermo Scientific) and incubated at 37°C for 24 h along with Anaerogen sachets and an anaerobic indicator (Thermo Scientific), which were placed in an anaerobic culture jar (BD BBL GasPak 100 jar, BD BBL) to maintain an anaerobic environment. These cultures were then streaked onto Tryptone Soya agar (TSA) plates (Thermofisher Scientific, Oxoid) supplemented with 5% sheep’s blood to ensure the viability and purity of the cultures.

### Antibiotic susceptibility testing (AST)

All antibiotic susceptibility assays were performed using cation-adjusted Mueller Hinton broth (CAMHB, Oxoid) in 96-well plates. The starting concentrations for ampicillin, gentamicin, meropenem, and tetracycline were 64 mg/L, 250 mg/L, 12.5 mg/L, and 250 mg/L, respectively. A 2-fold serial dilution was used to dilute the antibiotics. The starting bacterial optical density at 560 nm was 0.05, equivalent to 0.5 McFarland turbidity standards. The plates were incubated at 37°C for 20 h. All measurements were performed in triplicates. The resistance/susceptible status of antibiotics determined was interpreted according to reference breakpoints. Gentamicin was used as a negative control antibiotic, as anaerobes are inherently resistant to aminoglycoside, an oxygen-dependant antibiotic (52).

### Spore quantification

The overnight cultures (2%) were transferred to a 5 mL TGB supplemented with theobromine to 0.01% final concentration in 14 mL round bottom tubes, followed by incubation for 24 h at 37°C, under anaerobic conditions. For spore quantification, the culture was heated at 75–80°C for 10 min to kill the vegetative bacteria and activate the spores, cooled in water at room temperature for 30 min, and subsequently incubated at 40°C for 30 min, for spore germination. Aliquots from various isolates were plated on brain heart infusion (BHI) agar, and the heat-resistant spores were counted. The spore formation was also quantified using the anaerobic GVA protocol, by transferring 50 µL from each sample to 96-well plates and proceeding with the procedure (see GVA in materials and methods section).

### GVA

#### Embedding solution preparation

The preparation protocol was started ~40 min prior to the embedding of the sample. In a sterilized bottle, 0.66 g of agarose was combined with 100 mL of growth medium (TGB), resulting in a 0.66% wt/vol solution. The subsequent mixing with the sample results in a final concentration of 0.5% agarose, which has an approximate pore size of 600 nm. For *C. perfringens* selective agar preparation, we added 2.3 g of perfringens agar base (Oxoid CM0587) to 100 mL sterilized ddH2O. Sodium metabisulphite and ammonium ferric citrate are used in the media as indicators of sulfite reduction by *C. perfringens*, resulting in black colonies. The cap was adjusted to release steam before heating the agarose mixture in the microwave until it dissolved completely. Thorough swirling ensured the absence of any residual undissolved agarose. Alternatively, media could be subjected to autoclaving, followed by placement in a warm bath for extended storage. The melted agarose was placed in a warm bath for ~40 min to reach equilibrium.

#### Bacterial dilution and preparation for embedding

In the subsequent step, 50 µL from each treated condition, with a microbial load of <10^7^ CFUs/mL, should be placed in separate wells of a 96-well plate. For checking the viability/count of treated wells, it is preferable to perform the dilution step. However, if the aim is to check for MBC, the dilution step could be skipped to save time, and BG is used to increase the colonies' contrast.

#### Bacterial embedding

First, the agarose was carefully taken out from the heated bath and transferred into a storage container. To slow down the agarose gelling process, the reservoir was placed in a warm bead bath at 50°C. Gelling times were affected by agarose concentration. Higher concentrations led to faster gelling. Thus, it is recommended to optimize the timing, which will be tailored to the specific experimental system. A total of 150 µL of liquid media mixed with agarose was swiftly added to the wells containing the initial 50 µL samples. This was done using a 12-channel pipette. The mixing process was gently repeated 2–5 times to ensure everything was well combined. After mixing, a slow aspiration of 150 µL was performed. To avoid bubbles, we controlled the pipette speed and placed the tips carefully at the well’s base. The tips were briefly placed in an ice bath for approximately 10 s to avoid any embedding leakage. To remove any aggregates, the pipette tips were gently tilted and run along the surface of the ice after immersing them in an ice bath. The prepared tips were allowed to solidify at room temperature for approximately 30 min before being placed into an anaerobic jar. Inside the jar, anaerobic sachets (Thermo Scientific, catalog number: AN0035A), and an anaerobic indicator (Thermo Scientific Oxoid Resazurin Anaerobic Indicator, Catalog number: R65055) were added. This whole setup was then kept at 37°C overnight to create the ideal conditions for microbial growth. Colony positions were measured the following day. Tips can be kept at room temperature for several days with no appreciable change in the colony size.

#### Tip imaging and bacterial counting

After retrieving the overnight incubated tips, it is advised to wait for 10 min at room temperature before imaging to let them cool down. Imaging is performed using a custom instrument. Comprehensive assembly guidelines, a detailed inventory of components, and a schematic representation of the circuit governing the optical box’s operations can be readily accessed (https://www.colorado.edu/research/geometric-viability-assay/geometric-viability-assay-gva). A mirrorless commercial camera, specifically the Canon EOS RP, paired with a 1:1 macro lens from Canon with specifications of f/2.8 aperture and 100 mm focal length, was employed to capture images of superior quality capable of distinguishing even the minutest colonies. Through this camera lens setup, we calculated a pixel size of 6.6 µm, ensuring fine resolution. To position the tips within the lightbox, a 12-channel P200 pipette was employed. This apparatus allows for efficient imaging of a standard experiment with 96 tips in around 7 min.

We deployed here another imaging procedure using the White *Trans* Illumination setting of Chemidoc/Geldoc with 590/110 filter. The pipette tips were placed on the white tray (Bio-Rad) using a 12-channel P200 pipette, then the imaging field was adjusted based on the twelve tips’ dimensions. Output images were further processed using MATLAB (Mathworks, R2021b) to render it compatible with the GVA app (Code 1).

The analysis of tips' images includes two steps: (i) segmentation of pipette tips, where the multiple tips in a single image are split into separate tips for counting. (ii) Semi-automated colony segmentation, where the user could select one of five segmentation models according to the CFU distribution. The first model offers full tip segmentation for low dense tips, whereas the second segmentation model zooms into 1/20^th^ of the tip size for highly populated tips. The user is able to intervene by adding the missed colonies or removing the faulty ones. Then, the bacterial count (CFU/mL) of each tip, is stored in a CSV file, tagged to each well ID.

## SUPPLEMENTAL MATERIAL

Supplemental figures spectrum.02706-24-s0001.docx Fig. S1 and S2.

Supplemental data spectrum.02706-24-s0002.txt MATLAB code to process images from the gel imager, making them compatible with the GVA app for colony counting.

Table S1 spectrum.02706-24-s0003.docx Advantages and disadvantages of several viability methods for anaerobes.

ASM does not own the copyrights to Supplemental Material that may be linked to, or accessed through, an article. The authors have granted ASM a non-exclusive, world-wide license to publish the Supplemental Material files. Please contact the corresponding author directly for reuse.
