# Supplementary material for: A high-throughput anaerobic method for viability assays
Source: Microbiol Spectr. 2025 Mar 5;13(4):e02706-24. doi: 10.1128/spectrum.02706-24 (PMC11960071; doi:10.1128/spectrum.02706-24)
Supplement: Supplemental figures — Fig. S1 and S2. [file spectrum.02706-24-s0001.docx]

*
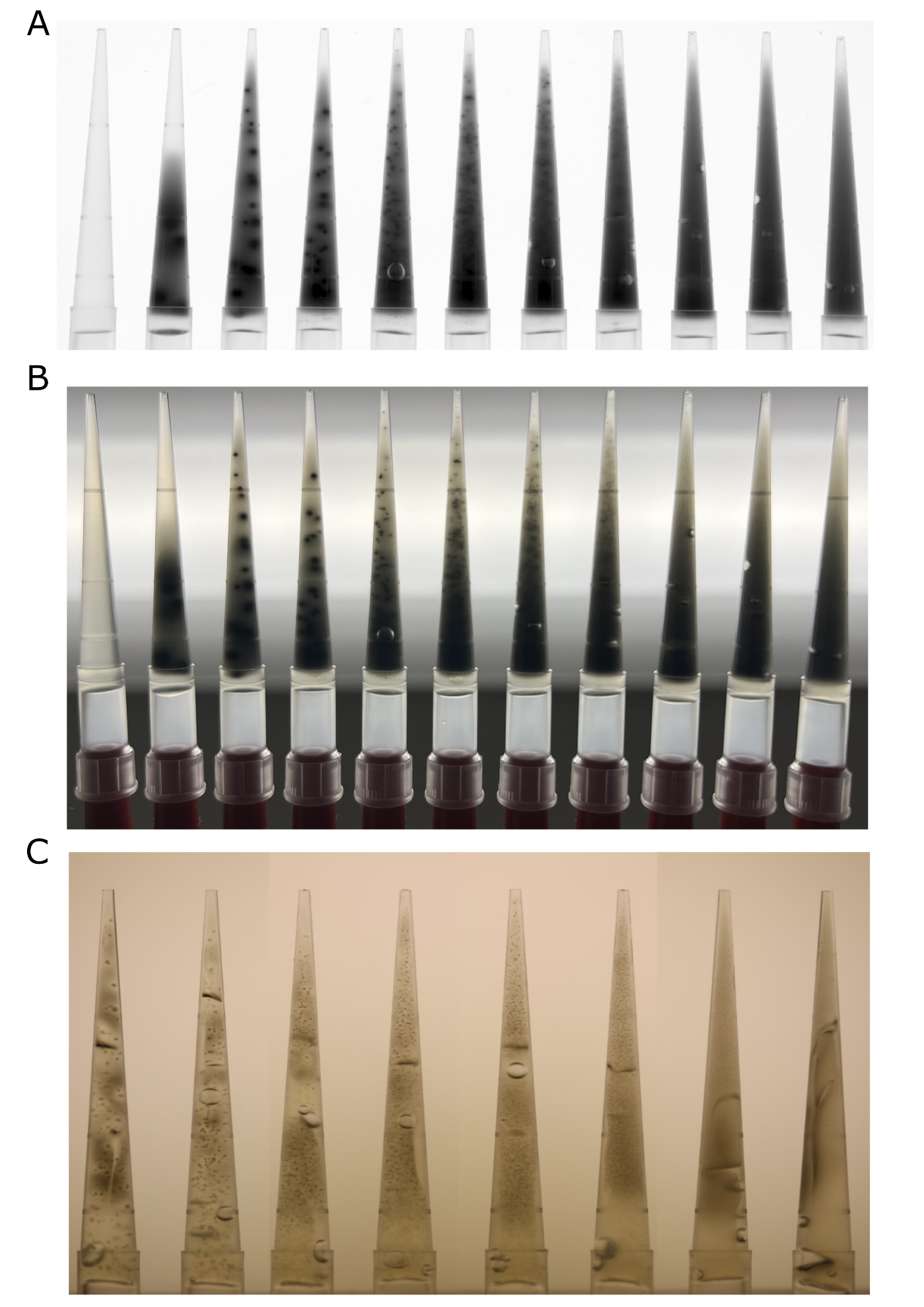
*

Figure S1. GVA. Imaging of Clostridium perfringens at different concentrations in tips for GVA with selective perfringens agar by various imaging systems: (a): the Chemidoc; (b): iPhone 13 camera. The tips were imaged in front of the white lamp; (c): the GVA imaging box. The opacity of the background has improved by imaging the tips using the GVA imaging box, which employ a LED light box with a DSLR camera.

*
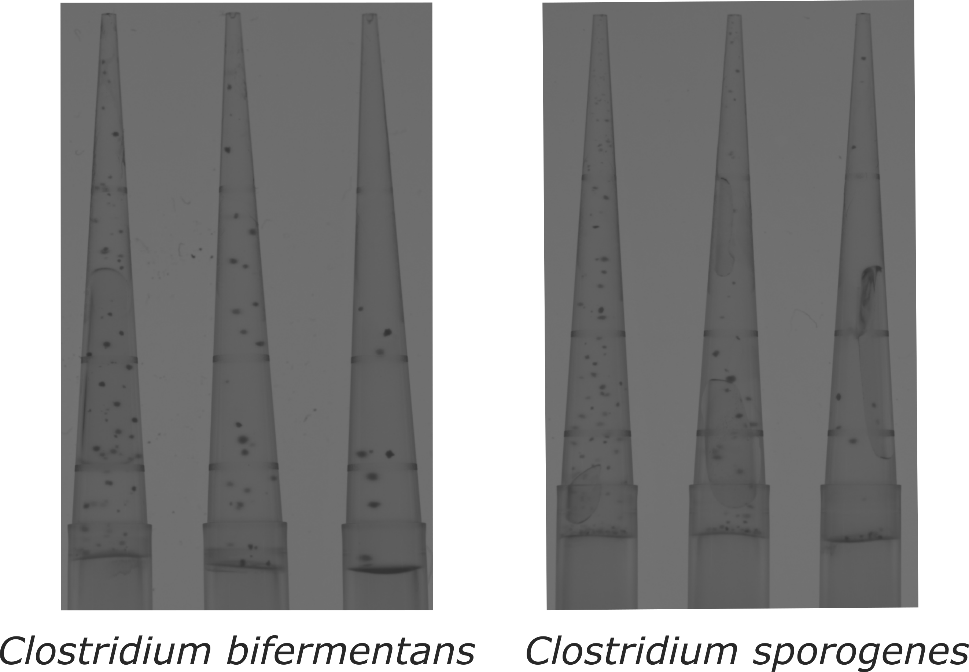
*

Figure S2. Imaging of Clostridium bifermentans and Clostridium sporogenes at different dilutions in tips for GVA. Bacteria were embedded in 150 μL TGB with 0.5% agarose in P200 pipette tips. GVA pipette tips were imaged using White Trans Illumination mode of ChemiDoc with 590/110 filter.
