# Supplementary material for: A high-throughput anaerobic method for viability assays
Source: Microbiol Spectr. 2025 Mar 5;13(4):e02706-24. doi: 10.1128/spectrum.02706-24 (PMC11960071; doi:10.1128/spectrum.02706-24)
Supplement: Table S1 — Advantages and disadvantages of several viability methods for anaerobes. [file spectrum.02706-24-s0003.docx]

*Table S1. Advantages and disadvantages of several viability methods for anaerobes.*

| Method | Advantages in anaerobes | Disadvantages in anaerobes | References for application in anaerobes |
| --- | --- | --- | --- |
| Plate-based Colony Forming Unit (CFU) Assay | - Well-established and widely used. - Allows for the assessment of colony morphology. | - Labor-intensive and time-consuming due to the need for serial dilutions and plating. - Requires a large number of consumables, such as agar plates and media. - Limited throughput. | (1, 2) |
| Resazurin Reduction Assay | - High throughput and easy to perform. - Measures metabolic activity in real-time. | - Metabolic activity does not always correlate with viability. - Fluorescent signals can be affected by certain compounds. | (3) |
| Optical density (OD) | - Suited for experiments requiring continuous growth curve analysis. - Can be automated for high-throughput screening. | - OD may not correlate with viable cell counts. - Sensitivity is confined to bacterial concentrations between 10⁸ to 10¹⁰ per mL. | (4) |
| Flow Cytometry | - Provides rapid, quantitative results. - Can differentiate live, dead, and damaged cells. | - Requires specialized and expensive equipment. - Involves complex sample preparation and data analysis. | (5) |
| Nucleic acid-based methods (eg. PCR, LAMP, EMA/PMA-qPCR) | - High sensitivity and specificity. - Provides quick results compared to culture-based methods. | - Does not differentiate between live and dead cells unless combined with viability dyes (e.g., EMA or PMA). - Requires expensive equipment and reagents. - Requires sample-specific optimization. | (6, 7) |
| Microfluidic chips | - Ultra-low sample and reagent volumes. - Capable of monitoring live/dead cell ratios in real time. | - Requires specialized fabrication equipment. - Challenging to maintain anaerobic conditions. - Initial setup costs are high. | (8) |
| Microscopy | - Can differentiate live and dead bacteria when proper dyes are used. - High sensitivity and specificity. | - Requires specialized equipment and expertise. - Limited throughput. - Sample preparation can be complex and time-consuming. | (9) |
| Geometric Viability Assay (GVA) | - Cost-effective and utilizes minimum consumables. - Enables high-throughput viability measurements. | - Requires proper visualization of tips to quantify the viable colonies, especially in dense samples. - Difficult to assess the morphology of colonies at high density. | This study |

References:

1. Alves J, Sargison FA, Stawarz H, Fox WB, Huete SG, Hassan A, McTeir B, Pickering AC. 2021. A case report: Insights into reducing plastic waste in a microbiology laboratory. Access Microbiology 3.

2. Pal R, Seleem MN. 2023. Antisense inhibition of RNA polymerase α subunit of *Clostridioides difficile*. Microbiol Spectr 11:e01755-23.

3. Simó C, Fornari T, García-Risco MR, Peña-Cearra A, Abecia L, Anguita J, Rodríguez H, García-Cañas V. 2022. Resazurin-based high-throughput screening method for the discovery of dietary phytochemicals to target microbial transformation of l-carnitine into trimethylamine, a gut metabolite associated with cardiovascular disease. Food Funct 13:5640-5653.

4. Müller P, de la Cuesta-Zuluaga J, Kuhn M, Baghai Arassi M, Treis T, Blasche S, Zimmermann M, Bork P, Patil KR, Typas A, Garcia-Santamarina S, Maier L. 2023. High-throughput anaerobic screening for identifying compounds acting against gut bacteria in monocultures or communities. Nat Protoc doi:10.1038/s41596-023-00926-4.

5. Tracey H, Coates N, Hulme E, John D, Michael DR, Plummer SF. 2023. Insights into the enumeration of mixtures of probiotic bacteria by flow cytometry. BMC Microbiol 23:48.

6. Song Y. 2005. PCR-based diagnostics for anaerobic infections. Anaerobe 11:79-91.

7. Mauerhofer L-M, Pappenreiter P, Paulik C, Seifert AH, Bernacchi S, Rittmann SKMR. 2019. Methods for quantification of growth and productivity in anaerobic microbiology and biotechnology. Folia Microbiol (Praha) 64:321-360.

8. Wang Z, Zhu T, Simpson David J, Gänzle Michael G. 2022. Supercharged MPNs? Automated determination of high-throughput most probable number (htMPN) using chip-based 3D digital PCR. Appl Environ Microbiol 88:e00822-22.

9. Liang D, Cui X, Li M, Zhu Y, Zhao L, Liu S, Zhao G, Wang N, Ma Y, Xu L. 2023. Effects of sporulation conditions on the growth, germination, and resistance of *Clostridium perfringens* spores. Int J Food Microbiol 396:110200.
